# Metabolomics and Transcriptomics in Legumes Under Phosphate Deficiency in Relation to Nitrogen Fixation by Root Nodules

**DOI:** 10.3389/fpls.2018.00922

**Published:** 2018-07-11

**Authors:** Mostafa Abdelrahman, Magdi A. El-Sayed, Abeer Hashem, Elsayed Fathi Abd_Allah, Abdulaziz A. Alqarawi, David J. Burritt, Lam-Son Phan Tran

**Affiliations:** ^1^Arid Land Research Center, Tottori University, Tottori, Japan; ^2^Department of Botany, Faculty of Science, Aswan University, Aswan, Egypt; ^3^Botany and Microbiology Department, College of Science, King Saud University, Riyadh, Saudi Arabia; ^4^Mycology and Plant Disease Survey Department, Plant Pathology Research Institute, Agricultural Research Center, Giza, Egypt; ^5^Plant Production Department, College of Food and Agriculture Sciences, King Saud University, Riyadh, Saudi Arabia; ^6^Department of Botany, University of Otago, Dunedin, New Zealand; ^7^Institute of Research and Development, Duy Tan University, Da Nang, Vietnam; ^8^Stress Adaptation Research Unit, RIKEN Center for Sustainable Resource Science, Yokohama, Japan

**Keywords:** legumes, metabolomics, transcriptomics, phosphate deficiency, nitrogen fixation

## Abstract

Phosphate (P_i_) deficiency is a critical environmental constraint that affects the growth and development of several legume crops that are usually cultivated in semi-arid regions and marginal areas. P_i_ deficiency is known to be a significant limitation for symbiotic nitrogen (N_2_) fixation (SNF), and variability in SNF is strongly interlinked with the concentrations of P_i_ in the nodules. To deal with P_i_ deficiency, plants trigger various adaptive responses, including the induction and secretion of acid phosphatases, maintenance of P_i_ homeostasis in nodules and other organs, and improvement of oxygen (O_2_) consumption per unit of nodule mass. These molecular and physiological responses can be observed in terms of changes in growth, photosynthesis, and respiration. In this mini review, we provide a brief introduction to the problem of P_i_ deficiency in legume crops. We then summarize the current understanding of how P_i_ deficiency is regulated in legumes by changes in the transcriptomes and metabolomes found in different plant organs. Finally, we will provide perspectives on future directions for research in this field.

## Introduction

**FIGURE 1:**
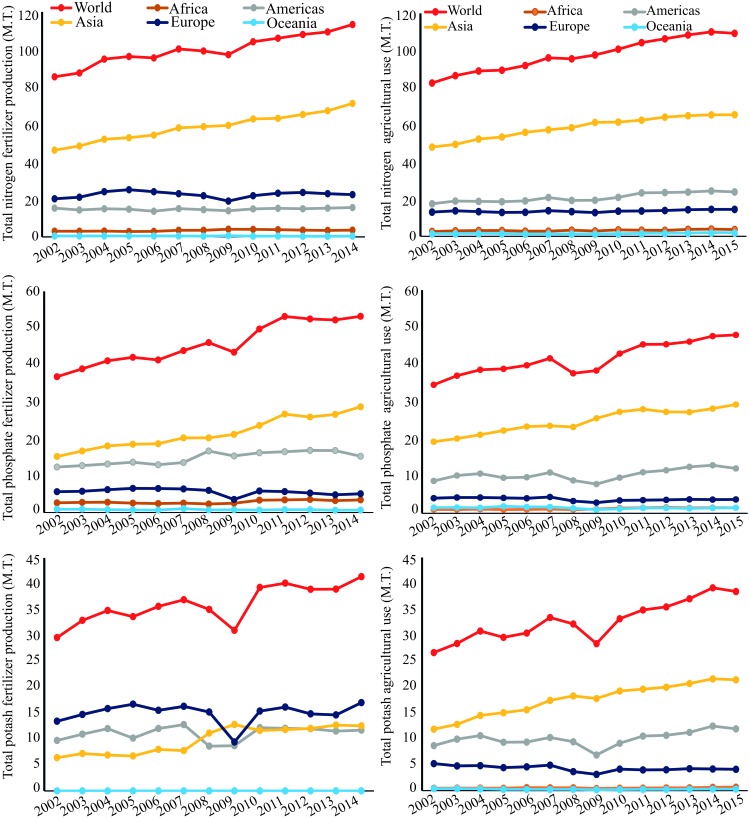
World nitrogen, phosphate (P_2_O_5_), and potash (K_2_O) fertilizer production from 2002 to 2014, and their agricultural use from 2002 to 2015 in millions of tonnes (M.T.), according to FAO (www.fao.org, accessed 2018).

Phosphorus(P) is a crucial element required for plant growth and development, playing a pivotal role in a diverse array of cellular processes, including photosynthesis, energy production, redox reactions, symbiotic nitrogen (N_2_) fixation (SNF), and carbohydrate metabolism (Sulieman and Tran, 2015; Kleinert et al., 2017). However, more than 30–40% of the world’s arable soils have low P contents, and by 2050 rock phosphate (P_i_) reserves, the most inexpensive form of P for fertilization of agricultural soils, may be exhausted (Vance et al., 2003; Herrera-Estrella and López-Arredondo, 2016; Kleinert et al., 2017). Uptake of P_i_ from some soils can be problematic for plants due to slow P_i_ diffusion rates and the formation of insoluble P_i_ complexes with cations, especially iron and aluminum in acid weathered soils (Valentine et al., 2010; Castro-Guerrero et al., 2016). In several cropping systems, P_i_-containing fertilizers are applied frequently to soils to enhance P_i_ availability, and thus yield. With the increasing demand for food, P_i_ fertilizer demand has increased four- to five-fold in last few decades, and is expected to continue increasing^1^ (**Figure 1**). This fact combined with a significant increase in P_i_ fertilizer production will add further pressure on the limited P_i_ reserve in the coming years (**Figure 1**). The use of P_i_-containing fertilizers is a short-term solution to a much greater problem, as the real challenge for scientists and farmers is to deliver food with high nutritional quality using sustainable agricultural practices (Castro-Guerrero et al., 2016).

**FIGURE 2:**
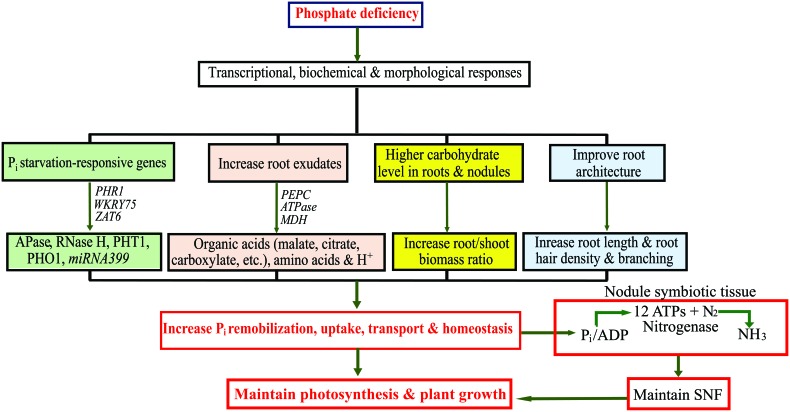
Schematic representation of transcriptional, biochemical and morphological changes in legumes under phosphate (P_i_) deficiency to increase P_i_ uptake and optimize P_i_ use efficiency. APase, ACID PHOSPHATASE; ATPase, Adenosine Triphosphatase; MDH, MALATE DEHYDROGENASE; PEPC, PHOSHOENOLPYRUVATE CARBOXYLASE; PHO1, PHOSPHATE TRANSPORTER 1; PHR1, PHOSPHATE STARVATION RESPONSIVE 1; PHT1, HIGH-AFFINITY PHOSPHATE TRANSPORTER 1; RNase H, RIBONUCLEASE H; SNF, symbiotic nitrogen fixation; ZAT6, ZINC FINGER OF *ARABIDOPSIS* 6.

Grain legumes are an essential source of nutrition and income for a large number of consumers and farmers worldwide (Kleinert et al., 2017; Abdelrahman et al., 2018). Legumes can create symbiotic relationships with N_2_-fixing rhizobia and arbuscular mycorrhizal fungi that facilitate the acquisition of nutrients; and thus, reduce the use of synthetic fertilizers, which is advantageous for sustainable agriculture (Considine et al., 2017; Valliyodan et al., 2017). SNF is an energetically expensive process, consuming ∼20 adenosine triphosphate (ATP) molecules for the production of two NH_3_ molecules (Thuynsma et al., 2014). Because of the requirement for large amounts of ATP for SNF, P_i_ deficiency is a critical constraint for efficient SNF in legumes. There is substantial evidence demonstrating that P_i_ deficiency can more severely affect the N:P ratio in legume tissues when compared with non-leguminous crops (Sulieman and Tran, 2015; Guo et al., 2016). Enhanced nutrient acquisition by SNF nodules formed by plants of P_i_-deficient soils is crucial for the efficient fixation of N_2_ (Magadlela et al., 2015; Lazali et al., 2017). Legumes have evolved conserved acquisition and internal transport strategies for P_i_ detected in P_i_-deficient soils in order to maintain nodule P_i_-homeostasis and enable efficient SNF (**Figure 2**). These include decreased plant growth rates, modification of carbon metabolism, increased secretion of organic anions and phosphatases, changes in root architecture, expansion of root surface areas, and enhanced expression of P_i_ transporters (Sulieman and Tran, 2015; Considine et al., 2017; Kleinert et al., 2017; Uhde-Stone, 2017). Because of the diminishing reserves of inexpensive P_i_ fertilizers, plant acclimation to P_i_ deficiency has become a topic of considerable interest to plant researchers. Below we present recent advances in the transcriptomic and metabolomic changes that occur in legumes in response to P_i_ deficiency, which is essential if we are to understand the complex systemic metabolic mechanisms plants use to adapt to P_i_ deficiency.

## A Legume Transcriptome Atlas Under P_i_ Deficiency

Next-generation sequencing (NGS) technologies have become essential tools to help understand the regulation of gene expression and the molecular basis of cellular responses that occur in plants exposed to biotic and/or abiotic stressors (Abdelrahman et al., 2015, 2017a,b; Miao et al., 2015; Liese et al., 2017; Nasr Esfahani et al., 2017). A number of transcriptome studies of leguminous plant species, including white lupin (*Lupinus albus*), common bean (*Phaseolus vulgaris*), soybean (*Glycine max*), chickpea (*Cicer arietinum*), and *Medicago truncatula*, grown under P_i_ deficiency have been conducted in the last several years (Hernández et al., 2007; O’Rourke et al., 2013; Liese et al., 2017; Nasr Esfahani et al., 2017; Zhang et al., 2017). RNAseq-based transcriptome profiling of nodules of *Sinorhizobium meliloti-*inoculated *M. truncatula* plants grown under P_i_ deficiency has shown a strong down-regulation in the expression of genes encoding NODULE-SPECIFIC CYSTEINE-RICH peptides, LEGHEMOGLOBIN and NICOTIANAMINE SYNTHASE-LIKE PROTEIN, compared with nodules of control plants grown under P_i_-replete conditions (Liese et al., 2017). The down-regulation of these genes disturbs normal cellular iron distribution, restricts the supply of oxygen for respiration and eventually lowers nitrogenase activity in nodules (Liese et al., 2017). This potential disruption of normal nodule metabolism caused by P_i_ deficiency greatly reduces SNF efficiency in legumes (Liese et al., 2017). In addition, a reduction in shoot and nodule dry matter, and tissue P_i_ levels was observed in P_i_-deficient *M. truncatula* plants relative to P_i_-replete control plants, indicating that P_i_ deficiency can severely limit legume growth and potential crop yields (Liese et al., 2017). However, while *S. meliloti*-inoculated *M. truncatula* plants grown under P_i_ deficiency had much lower stem and root tissue P_i_ concentrations compared with control (P_i_-sufficient) plants, nodule P_i_ levels in P_i_-deficient plants were maintained at relatively high levels and did not show the considerable loss of P_i_ as observed for stems and roots (Liese et al., 2017). Nasr Esfahani et al. (2017) examined transcriptome changes in the nodules of P_i_-deficiency-more-susceptible *Mesorhizobium mediterraneum* SWRI9-(*Mm*SWRI9)-chickpea and P_i_-deficiency-less-susceptible *M. ciceri* CP-31-(*Mc*CP-31)-chickpea associations under P_i_-deficient and -sufficient conditions. The transcriptome profiles of these interactions showed that many genes related to several key cellular processes and metabolic pathways namely transcriptional regulation, detoxification, nodulation, ion/nutrient transport, and P_i_ signaling and remobilization were differentially expressed in *Mm*SWRI9-induced nodules relative to *Mc*CP-31-induced nodules (Nasr Esfahani et al., 2017). Changes in the expression of P_i_ starvation-related genes are likely to help improve acquisition and transport of P_i_ in the *Mm*SWRI9-chickpea association; and thus maintenance of the sufficient SNF capacity under P_i_-deficient conditions (Nasr Esfahani et al., 2017). The above observations indicated that changes in legume transcriptomes under P_i_ starvation are mostly associated with facilitating P_i_ solubilization, acquisition and transportation into nodules, which are significant sinks for P_i_, contributing to more efficient SNF and therefore higher plant productivity.

Although most studies to date have focused on nodule transcriptomes, exploring the transcriptomes of other plant tissues is also important, as these may provide valuable insight into P_i_ deficiency acclimation mechanisms in legumes. For example, transcriptome analysis of leaves and roots (combined cluster and normal roots) of white lupine plants grown under P_i_-deficient or -sufficient conditions identified 1,342 and 904 differentially expressed genes, respectively, in response to P_i_ deficiency (O’Rourke et al., 2013). In leaves, the most highly expressed transcripts were involved in amino acid metabolism, tetrapyrrole synthesis, photosynthesis, carbohydrate catabolism, and flavonoid biosynthesis; whereas in roots the most highly expressed transcripts were involved in sugar/nutrient signaling and transport, lignin biosynthesis, phospholipid and carbohydrate metabolism, and amino acid synthesis. Interestingly, 12 transcripts identified in the above study were commonly induced by low-P_i_ stress across three species, white lupine, *Arabidopsis*, and potato (*Solanum tuberosum*), making them excellent candidates to investigate responses to P_i_ starvation in plants (O’Rourke et al., 2013). Among these 12 transcripts, three transcripts that were highly up-regulated in P_i_-deficient lupine plants compared with P_i_-sufficient ones (O’Rourke et al., 2013) encode SPX domain-containing proteins [RECOMBINANT *Saccharomyces cerevisiae* PROTEIN (SYG1)/PHOSPHATASE(PHO81)/XENOTROPIC and POLYTROPICRETROVIRUS RECEPTOR 1(XPR1)] that are essential regulators involved in P_i_ homeostasis and signaling responses to P_i_ deficiency (Chiou and Lin, 2011; Secco et al., 2012). In addition, P_i_ solubilization- and transport-related genes, encoding PHOSPHATE TRANSPORTER 1, PHOSPHOLIPASE, PYROPHOSPHATASE, PURPLE ACID PHOSPHATASE, and MONOGALACTOSYLDIACYLGLYCEROL SYNTHASE, were also up-regulated in lupine P_i_-deficient relative to P_i_-sufficient plants (O’Rourke et al., 2013). A recent study by Zhang et al. (2017) provided transcriptome datasets obtained from the roots and leaves of soybean plants grown under P_i_-deficient and -sufficient conditions, and showed a significant role for the acid phosphatase*-*encoding gene *GmACP1* in regulating P_i_ use efficiency in soybean. These results were in agreement with a previous finding by the same research group (Zhang et al., 2014), who used a genome-wide association study of 192 soybean accessions to identify a quantitative trait locus (QTL) on soybean chromosome 8, namely *qPE8*, which was associated with improvement of soybean P_i_ use efficiency under P_i_ starvation. This *qPE8* QTL contained the candidate genes *Glyma08g20700*, *Glyma08g20710*, and *Glyma08g20800*, *Glyma08g20820*/*GmACP1* and *Glyma08g20830*, which encode CALCINEURIN B, PHOSPHOLIPASE D and putative PHOSPHATASES, respectively. *Glyma08g20820*/*GmACP1* was up-regulated under P_i_ deficiency; however, the transcript levels of the remaining genes were not changed (Zhang et al., 2014). In addition, overexpression study of *GmACP1* using hairy-root transformation showed that the transgenic hairy roots displayed a 2.3-fold increase in acid phosphatase activity and an 11.2–20.0% higher P_i_ use efficiency relative to wild-type plants under P_i_ starvation (Zhang et al., 2014).

Transcriptome analysis of wild legumes is also critical to aid in understanding the differences that exist between domesticated legumes and their wild progenitors (Abdelrahman et al., 2018). This could also help provide a better understanding of the P_i_ stress adaptation mechanisms present in wild legumes. In addition, transcriptome correlation analyses between different legume species under P_i_ deficiency may provide crucial information about the conserved P_i_ deficiency-responsive genes, which could be used as molecular markers for screening for low P_i_- tolerant/susceptible cultivars or genetic engineering to enhance the growth and productivity of crop plants grown on low P_i_ soils.

## Transcriptional Regulation and MicroRNA Under P_i_ Deficiency

Plants adapt to P_i_ starvation by an array of molecular responses in which transcription factors (TFs) are key components in the regulation of these processes (Jain et al., 2012). The transcriptional regulations of the P_i_ starvation responses have been extensively studied in other plant species; however, these important processes have not much investigated yet in legumes. Four TF-encoding genes *BASIC HELIX-LOOP-HELIX 32* (*BHLH32*), *WRKY75*, *PHOSPHATE STARVATION RESPONSIVE 1* (*PHR1*), and *ZINC FINGER OF ARABIDOPSIS 6* (*ZAT6*) involved in P_i_ starvation signaling have been identified in *Arabidopsis* (Valdés-López and Hernández, 2008). *AtPHR1* and its orthologs from rice (*Oryza sativa*, *OsPHR1* and *OsPHR2*) are regarded as the key positive regulators controlling plant transcriptional responses to P_i_ deficiency (Rubio et al., 2001; Zhou et al., 2008). The overexpression of *AtPHR1* induced P_i_-responsive genes involved in P_i_ remobilization (*ACID PHOSPHATASES and RIBONUCLEASE H*), transport [*HIGH*-*AFFINITY PHOSPHATE TRANSPORTER 1* (*PHT1*), *PHOSPHATE TRANSPORTER 1*(*PHO1*)] and homeostasis (*miRNA399* and *At4*), in addition to genes involved in anthocyanin biosynthesis (Valdés-López and Hernández, 2008). Hernández et al. (2007) reported 17 TF-encoding genes differentially expressed in common bean roots under P_i_ deficiency. Of these genes, *TC2883 MYB* gene was highly induced under P_i_ starvation and exhibited 63% homology to *AtPHR1*, suggesting an important role of PHR1 in common bean response to P_i_ deficiency. Likewise, the *Arabidopsis At4* plays a significant role in translocation of P_i_ from roots to shoots, and its ortholog from *M. truncatula*, the *Mt4*, showed strongly induced expression in roots under P_i_ deficiency (Valdés-López and Hernández, 2008). *WKRY75* and *ZAT6* are also up-regulated under P_i_ starvation, and both two genes are implicated in P_i_ remobilization, transport, and homeostasis as well as root architecture. In contrast, *BHLH32* is down-regulated under P_i_ deficiency, and its role in modification of root architecture has been proposed (Chen et al., 2007).

Overexpression of the rice *osa-miR827* and *Arabidopsis miR399*/*miRNA399* that target the SPX-MAJOR FACILITATOR SUPERFAMILY (MFS) protein-encoding genes and the P_i_ transporter genes, respectively, drastically impacts P_i_ homeostasis and accumulation in transgenic plants (Wang et al., 2012; Chen et al., 2017). During P_i_ starvation, *miR399*/*miRNA399* supresses its target gene *PHO2* and allows sufficient transcript of *PHT1* accumulated in the membrane of P_i_-starved transgenic plants, thereby increasing P_i_ acquisition (Franco-Zorrilla et al., 2007). Recently, Chen et al. (2017) demonstrated a crucial role of the *TamiR167a* in mediating tobacco (*Nicotiana tabacum*) growth and adaptation to P_i_ starvation via regulation of various biological processes, including P_i_ acquisition and reactive oxygen species homeostasis. Thus, distinct miRNAs are also important regulators in mediating the plant response to P_i_ stress as well.

## Legume Metabolome Profile Under P_i_ Deficiency

The development of crop plants that are able to produce good yield on nutrient-deficient soils requires an in-depth knowledge of physiological and biochemical processes that allow plants to survive under these stressful conditions. Integrated transcriptomic and metabolomic studies can aid in obtaining this knowledge (Hirai et al., 2004; Last et al., 2007; Hernández et al., 2009; Saito, 2013; Abdelrahman et al., 2014, 2015, 2017c,d; Jin et al., 2017). Plant metabolites are synthesized by numerous proteins/enzymes encoded in the plant genome, and integration of gene expression atlas with targeted/non-targeted metabolite profile is an innovative approach to identify gene-to-metabolite associations/networks (Hirai et al., 2004; Saito, 2013; Abdelrahman et al., 2017d). The use of metabolic profiling has been quite limited for legume crops, but this approach has recently been applied to help understand the metabolic changes associated with legume-rhizobial symbiosis. Symbiotic N_2_-fixing bacteria secrete lipo-chitooligosaccharide signaling molecules, also known as Nod factors, upon perception of isoflavonoids and flavonoids secreted by legume roots (Zhang et al., 2012). The Nod factors are perceived by their receptors on the plasma membranes of root cells of leguminous plants, which then activate signaling processes within the nucleus and cytoplasm of target cells (Zhang et al., 2012). Untargeted metabolite profiling of the extracts of *M. truncatula* seedlings treated with rhizobial lipo-chitooligosaccharide molecules has shown a significant decrease in oxylipin-related compounds in *M. truncatula*. Oxylipins are precursors of the jasmonic acid biosynthesis pathway, and both oxylipins and jasmonic acids inhibit Nod factor signaling, suggesting that these oxylipin-related compounds act as negative regulators of the early stages of symbiosis (Zhang et al., 2012).

In an early study, Hernández et al. (2009) used integrated non-targeted metabolite profiling and transcriptome analysis to identify changes in the roots and nodules of common bean plants inoculated with *Rhizobium tropici* and grown under P_i_-deficient and -sufficient conditions. They showed clear metabolic differences between plants grown under these two contrasting conditions. Integrative analysis of nodule transcriptome and metabolome allowed the authors to identify 13 metabolites that could be assigned to repressed or induced pathways in response to P_i_ deficiency. Of these 13 P_i_ starvation-responsive metabolites, a reduction in N metabolism-related metabolites, including spermidine, putrescine, urea, glycine, serine, glutamine, and threonine, was detected in nodules of P_i_-deficient common bean plants relative to that of P_i_-sufficient ones, and this change might contribute to a decrease in SNF efficiency (Hernández et al., 2009). In addition, P_i_-deficient common bean roots show reduced levels of organic acids like tartaric acid and 2,4-dihydroxybutanoic acid, due to the secretion of these organic acids into the rhizosphere (Hernández et al., 2009). In contrast, alteration of carbon (C) metabolism in P_i_-deficient common bean results in lower and higher carbohydrate levels in the shoots and roots, respectively, thereby contributing to the increased root/shoot biomass ratio and altering root morphology (Hernández et al., 2009). Similarly, Nasr Esfahani et al. (2016) showed lower SNF efficiency and decreased P_i_ level in the P_i_-deficiency-more-susceptible *Mm*SWRI9-chickpea nodules than the P_i_-deficiency-less-susceptible *Mc*CP-31-chickpea nodules under Pi deficiency, which was evident by significant differences in C and N metabolism-related metabolites. For example, in *Mc*CP-31-inoculated plants, P_i_ deficiency increased total level of identified sugars by 68.8%, whereas that remained unchanged in *Mm*SWRI9-induced nodules (Nasr Esfahani et al., 2016). In addition, P_i_ deficiency induced a remarkable increase in total level of organic acids in *Mc*CP-31-nodulated roots, whereas it decreased that in *Mm*SWRI9-nodulated roots (Nasr Esfahani et al., 2016). These results revealed the existence of crosstalk among various signaling pathways involved in regulation of *Mesorhizobium*-chickpea adaptation to P_i_ deficiency, in-depth understanding of which at genetic level will be useful for genetic engineering of chickpea cultivars and other leguminous crops that can sustain efficient SNF under P_i_ deficiency. C and N metabolism is essential for SNF, and is a significant determinant of plant and nodule responses to P_i_ starvation (Kleinert et al., 2017). Some studies have shown that even under P_i_ deficiency, plant nodules continue to act as very strong nutrient sinks for C in order to maintain SNF, and underground biomass often continues to increase even at the expense of whole plant growth (Thuynsma et al., 2014; Magadlela et al., 2015). Interestingly, for white lupine plants grown under P_i_-deficient and -sufficient conditions, no significant differences in the above and below ground biomass between P_i_-deficient and -sufficient plants were observed, nor were any large differences in resource allocation (N and P_i_) between the shoot and root/nodule systems (Thuynsma et al., 2014). However, white lupine plants produced more cluster root biomass, up to 24% of the root system under P_i_ deficiency; relative to approximately 5% increase of the root system with sufficient P_i_ supply. In contrast, less nodule biomass (up to 14% of the root system) was detected under P_i_ deficiency than (up to 20% of the root system) sufficient P_i_ supply. In addition, cluster roots exhibited a significant increase in P_i_ acquisition rates under deficient P_i_ than sufficient P_i_ conditions (Thuynsma et al., 2014). These results suggest that underground adaptations, rather than large changes in shoot/root biomass ratio, may underpin the ability of lupine plants to grow well on P_i_-deficient soils, as more cluster root biomass would result in an improved P_i_ uptake rate; and hence help maintain high P_i_ level in the nodules, consequently efficient SNF under P_i_ deficiency (Thuynsma et al., 2014).

A recent metabolite profiling study of common bean root exudates grown in liquid culture media supplemented with P_i_ concentrations ranged from 0 to 8 mg L^-1^ showed that the levels of some organic acids, nucleic acids, and amino acids were much higher in common bean root exudates under P_i_-deficient conditions than P_i_-sufficient ones (Tawaraya et al., 2014). On the other hand, levels of phosphate esters, including glucose-6-phosphate, fructose-6-phosphate, and fructose-1, 6-phosphate, were lower in P_i_-deficient relative to P_i_-sufficient conditions (Tawaraya et al., 2014). The increase in amino acid and organic acid levels in the root exudates changed the respiration rate and influenced microsymbiont community in the root nodules, improving SNF under P_i_ deficiency (Tawaraya et al., 2014). While relatively few metabolomic profiling studies of legume crops under P_i_ deficiency have been conducted to date, future studies on legumes using an integrated metabolomic-transcriptomic approach may provide valuable information on the metabolic reprogramming at molecular level, which is required by plants for better adaptation to P_i_ deficiency.

## Summary and Future Perspectives

Legume crops are widely cultivated in many semi-arid and tropical parts of the world where P_i_ deficiency poses severe threats to crop productivity. To sustain legume cultivation under deficient P_i_ conditions, crop improvement programs require innovative methods, such as an integrated approach of transcriptomics and metabolomics to gain in-depth understanding of how plants respond to P_i_ deficiency at the molecular level. This mini review provides an overview of several transcriptomic and metabolomic studies conducted for legumes grown under P_i_ deficiency and their potential to help understand how legume crops respond to P_i_ deficiency (**Figure 2**). Transcriptomics and metabolomics have generated gigabyte-size data sets that require specialized computational software and bioinformatic tools to analyze them. To aid with this, several transcriptomic and metabolomic databases have been created; e.g., the MedicCyc for *M. truncatula*^2^, which includes more than 250 pathways with related metabolites, enzymes and associated genes. Another database, the Soybean Knowledge Base (SoyKB)^3^ has also been constructed. This resource is not only useful for soybean translational genomics, but also for legume proteomics and metabolomics. Identification of candidate genes and metabolic pathways important for the adaptation of legumes to P_i_ deficiency could be used in future for the marker-assisted selection of P_i_-efficient genotypes. Characterizing the proteomes of legumes under P_i_ deficiency is also a significant task for the future. In addition, information generated from transcriptomics and metabolomics combined with information from other types of analyses, including reverse and forward genetic analyses, could lead to the long-elusive goal of improvement of N_2_ fixation in agronomically essential grain legumes grown under P_i_ deficiency.

## Author Contributions

MA and L-SPT conceived the idea. MA, ME-S, AH, EA_A, AA, DB, and L-SPT wrote the manuscript. All authors read and approved the final manuscript.

## Conflict of Interest Statement

The authors declare that the research was conducted in the absence of any commercial or financial relationships that could be construed as a potential conflict of interest. The reviewer AK and handling Editor declared their shared affiliation.

## Footnotes

1 www.fao.org

2 https://www.plantcyc.org/typeofpublication/mediccyc

3 http://soykb.org/
